# Complete Genome Sequence of Uropathogenic *Escherichia coli* Strain CI5

**DOI:** 10.1128/genomeA.00558-15

**Published:** 2015-05-28

**Authors:** Kurosh S. Mehershahi, Soman N. Abraham, Swaine L. Chen

**Affiliations:** a National University of Singapore, Singapore; b Genome Institute of Singapore, Singapore; c Duke–National University of Singapore Graduate Medical School, Singapore; d Duke University Medical Center, Durham, North Carolina, USA

## Abstract

*Escherichia coli* represents the primary etiological agent responsible for urinary tract infections, one of the most common infections in humans. We report here the complete genome sequence of uropathogenic *Escherichia coli* strain CI5, a clinical pyelonephritis isolate used for studying pathogenesis.

## GENOME ANNOUNCEMENT

Urinary tract infections (UTIs) are one of the most frequently encountered bacterial infections, with uropathogenic *Escherichia coli* (UPEC) responsible for more than 80% of community acquired infections (1). Although often characterized as self-limiting and amenable to antibiotic therapy, UTIs often recur, causing significant morbidity to individual patients (2). Recurrent UTIs further lead to potential public health concerns due to high antibiotic usage (3, 4). Studies of UPEC pathogenesis have revealed that intracellular infection of bladder epithelial cells is a key feature leading to bacterial survival, antibiotic resistance, and recurrent UTI (5–20). UPEC strain CI5 is a clinical pyelonephritis isolate (21) that has been used in many of these studies using both *in vitro* cell culture models and *in vivo* murine infection models (22, 23). These have examined the role of Toll-like receptor 4 (TLR4) (13), cyclic AMP (cAMP), and Ca^2+^ signaling during UPEC invasion into bladder epithelial cells and the subsequent epithelial cell response (12, 16, 24). Additional studies have used CI5 to understand kidney infection and renal nephropathy during *in vivo* infection in mice (25). The genome sequence of CI5 will thus serve as a useful resource for future studies into the infection cycle of this important human pathogen.

CI5 genomic DNA was sheared to a size of approximately 10 kbp using a g-Tube (Covaris). An SMRTbell library was prepared according to the manufacturer’s instructions, loaded with a MagBead bound library protocol, and sequenced using the P4-C2 chemistry on the PacBio RS II instrument (Pacific Biosciences) with a 180-min movie time. *De novo* assembly was performed with the Hierarchical Genome Assembly Process (HGAP3) in the SMRT Analysis suite version 2.3 using default parameters (26). In total, there were 249,158 reads and 822,531,331 nucleotides that passed filtering, representing an approximate coverage of 80× (based on the final assembly) and a preassembly mean read length of 8,815 bp.

UPEC CI5 harbors a single chromosome of 4,885,378 bp with a G+C content of 50.8% and a previously unknown plasmid (pCI5) of 207,265 bp with a G+C content of 47.3%. Annotations of the CI5 genome and plasmid were performed using the NCBI Prokaryotic Genome Annotation Pipeline (PGAAP) (27). The CI5 chromosome and plasmid together contain 4,879 protein coding sequences, as well as 22 rRNA and 88 tRNA genes. The finished genome sequence of UPEC CI5 and its newly discovered plasmid pCI5 will aid in precise genetic manipulation and thereby further improve the study of UPEC virulence.

### Nucleotide accession numbers. 

The complete sequences of the uropathogenic *E. coli* CI5 chromosome and plasmid have been submitted to GenBank under the accession numbers CP011018 and CP011019, respectively.
